# Factors Associated with Length of Hospital Stay among HIV Positive and HIV Negative Patients with Tuberculosis in Brazil

**DOI:** 10.1371/journal.pone.0060487

**Published:** 2013-04-12

**Authors:** Maria Jacirema Ferreira Gonçalves, Alaidistania A. Ferreira

**Affiliations:** 1 Post-Graduate Program in Health, Society, and Endemic Diseases in the Amazon, Amazonas Federal University/Para Federal University/Leonidas & Maria Deane Institute (Fiocruz Foundation), Manaus, Amazonas, Brazil; 2 Manaus Nursing School, Amazonas Federal University, Manaus, Amazonas, Brazil; 3 Leonidas & Maria Deane Institute (Fiocruz Foundation), Manaus, Amazonas, Brazil; Fundacion Huesped, Argentina

## Abstract

**Objective:**

Identify and analyze the factors associated to length of hospital stay among HIV positive and HIV negative patients with tuberculosis in Manaus city, state of Amazonas, Brazil, in 2010.

**Methods:**

Epidemiological study with primary data obtained from monitoring of hospitalized patients with tuberculosis in Manaus. Data were collected by interviewing patients and analyzing medical records, according to the following study variables age, sex, co-morbidities, education, race, income, lifestyle, history of previous treatment or hospitalization due to tuberculosis, treatment regimen, adverse reactions, smear test, clinical form, type of discharge, and length of hospital stay. The associated factors were identified through chi-square or *t-*Student test at a 5% significance level.

**Results:**

Income from 1 to 3 minimum wages (P = 0.028), pulmonary tuberculosis form (P = 0.011), negative smear test or no information in this regard (P = 0.014), initial 6-month treatment scheme (P = 0.029), and adverse drug reactions (P = 0.021) were associated to prolonged hospital stay in HIV positive patients.

**Conclusion:**

We found out that although there were no significant differences in the length of hospital stay in HIV positive patients, all factors significantly associated to prolonged hospital stay occurred in this group of patients. This finding corroborates other studies indicating the severity of tuberculosis in HIV patients, which may also contribute to lengthen their hospital stay.

## Introduction

In Brazil, after the implementation of chemotherapy, which showed excellent results regarding clinical improvement and healing, the treatment of tuberculosis (TB) is mainly provided through outpatient care; the costs are low and, in most cases, patient’s hospitalization isn’t necessary. TB treatment is free in the country and it should be provided at the primary health care unit which is nearest to the patient’s home, since the Brazilian health system is decentralized; thus, hospitalization is recommended only in unusual and severe cases, and it must be as short as possible [1].

Despite the changes in public health policy, eliminating isolation and confinement in hospitals and sanatoriums and returning the patient to his community, the number of hospital admissions remains high. The ratio of patients with TB treated in hospitals is much higher than expected in various Brazilian regions [2]. Elsewhere in the world, the acquired immunodeficiency syndrome (AIDS) has been a major health problem leading to hospitalization [3], [4], [5]; although there’re few studies on hospitalization due to TB and AIDS in the country, the TB/HIV co-infection accounts for 30.0% of co-morbidities in hospitalized patients in Rio de Janeiro [6]. In Brazil, there’re regional differences regarding TB, especially when taking into account the TB/HIV co-infection, but few studies on hospitalization due to TB have been conducted. A higher number of patients with TB/HIV is expected in hospitals [6], since HIV may increase the incidence and severity of TB and disseminate extrapulmonary and/or atypical TB forms [7].

Manaus, capital city of the state of Amazonas, Brazil, accounts for 51.7% of the state population [8]. In 2010, the city had a TB incidence rate of 93.2 cases per 100,000 inhabitants. Manaus accounted for 68.7% (1,582/2,303) of the newly notified TB cases in the state and 95.8% (432/451) of hospitalized patients due to this disease, according to data from the Brazilian Ministry of Health [9].

Portaria GM number 1,101, enacted on June 12, 2002, from the Brazilian Ministry of Health, sets up the parameters for calculating the hospitalization coverage. According to this Portaria, the phthisiology area employs the following formula: ((population×0.08)×(0.13)/100.

According to the Population Census, Manaus had 1,802,014 inhabitants in 2010; so, the estimated number of admissions to the phthisiology sector in the city would be around 187. Thus, there’s an excessive number of hospitalizations in Manaus. The Portaria mentioned above provides that for TB cases with extensive lesions the maximum length of hospital stay should be 25 days/year; periods over this are regarded as protracted hospitalization.

Several factors have been suggested as contributing factors to TB, but studies on hospitalization due to this disease are rare in Brazil: only one study addressing the length of hospital stay was found [2]. Therefore, this study aims to identify and analyze the factors associated to the length of hospital stay of patients with TB in Manaus, in 2010, according to the HIV status.

## Methods

This is an epidemiological follow-up study addressing patients with TB hospitalized in referral hospitals in Manaus, within the period from January to December 2010. The city has an area of 11.401 km^2^ and, then, its population was 1,802,525 inhabitants [8]. There’re 9 hospitals in the capital (both general and specialized ones) and two of them provide specialized child care [10].

The inclusion criteria were patients with TB, aged ≥15 years, both those previously diagnosed before admission and the new cases identified during hospital stay, according to the diagnostic standard adopted by hospital medical services. The exclusion criteria were: patients who didn’t agree to be interviewed; those unable to answer questions due to mental confusion or inability to provide all information requested; and patients aged <18 years who aren’t accompanied by the legal guardian.

Data were collected at the following hospitals:

1. Getulio Vargas University Hospital – medium-sized institution with 149 beds; it admits patients with co-morbidities, and, as a university hospital, there’re admissions which contribute to the investigation on TB;
2. Adriano Jorge Foundation Hospital – general hospital with 255 beds which is a referral hospital for adults with TB and has 17 beds in the phthisiology sector; and
3. Dr. Heitor Vieira Dourado Tropical Medicine Foundation of Amazonas – medium-sized referral hospital with 123 beds, a state reference for infectious diseases and, as a result, for TB/HIV co-infected patients.

These hospitals concentrate on admissions of adult patients with TB; generally, when admitted to other hospitals in Manaus, the patients are transferred to one of them.

Data collection was conducted through structured and pre-tested questionnaires, directly interviewing the patient or his/her proxy informant, with a subsequent survey on medical records at hospital discharge.

The study variables were: age, sex, co-morbidities (e.g. hypertension, diabetes, HIV/AIDS, lung disease, and other health problems), education, race, income, smoking, alcoholism, drug use, history of previous treatment or hospitalization due to TB, treatment regimen, adverse reactions, smear test, clinical form, type of discharge, and length of hospital stay.

A patient was regarded as HIV-infected when a screening HIV test through enzyme-linked immunoabsorbent assay (ELISA) was positive, followed by a confirmatory positive test. This confirmation must be obtained through a second blood sample, when a new ELISA and an indirect immunofluorescence test or a western blot was positive [11]. The HIV status was defined as positive or negative having the result registered in the medical records or the test result itself as a basis.

The race classification follows the guidelines of the Brazilian Institute of Geography and Statistics (IBGE). People are asked to classify themselves based on their race phenotype, thus, we adopted the terms black, yellow, white, brown, and indigenous [12].

Regarding the use of alcohol, we used the Cut down/Annoyed/Guilty/Eye-opener questionnaire (CAGE) [13], and the analysis regarded as positive patients those who scored two or more questions. The tobacco dependence degree, according to the Fagerström index, was categorized as mild, medium, and high [14]. Concerning the length of hospital stay, we took into account the admission and discharge dates, and when successive hospital admissions occurred within a 15-day interval, we recorded them as a single hospitalization. We analyzed through STATA (STATACORP 9.0, TX-USA).

We present data under the form of frequency and percentage regarding socio-economic, lifestyle, and clinical/epidemiological variables, according to the HIV infection status, stratified by ≤25 days vs. >25 days of hospital stay. This period was adopted in accordance with the Portaria GM number 1,101/2012, from the Ministry of Health, which provides, in its paragraph 3.3 (Parameters for the Calculation of the Average Hospital Stay Rate), that TB with extensive lesions is limited to a maximum of 25 days/year per hospitalization. Thus, over 25 days it’s regarded as “prolonged hospitalization”. For this analysis, we used the Pearson chi-square test or Fisher’s exact test. We also used the two-tailed *t*-Student test to compare mean differences between groups, performing a priori tests for equality of variances. For all tests, a P-value <0.05 was regarded as statistically significant.

This study was approved by the Ethics Committee of the Tropical Medicine Foundation, under the Protocol 1,960 (CAAE 0006.0.114.115-09).

## Results

From January to December 2010, 306 hospital admissions of patients aged ≥15 years, with TB were identified in Manaus; forty-seven (15.4%) patients weren’t interviewed: one was absent at the time of the interview; 7 didn’t agree to participate in the study; 36 died as soon as they were hospitalized; and three were undergoing confusion and/or hadn’t a companion able to provide information. There was no statistically significant difference regarding sex between the interviewed and not interviewed groups (P = 0.8). The mean age doesn’t differ regarding length of hospital stay between respondents and no respondents (P = 0.11). Although it wasn’t possible to interview all hospitalized patients, we collected data from their medical records, in order to avoid information loss.

The mean length of hospital stay was 28.2 days (standard deviation (SD) = 32.6), and no significant difference regarding the HIV status (HIV+ average length of hospital stay = 29.6, SD = 25.2; HIV− average length of hospital stay = 26.9, SD = 38.6) was observed. Out of the total study population, 152 patients were HIV+ (49.7%) and 154 were HIV− (50.3%). Stratifying 25 days as a cutoff point regarding the length of hospital stay, it seems that men with HIV+ tend to stay longer in hospital, although it isn’t statistically significant (P = 0.072). HIV+ patients with an income from one to three minimum wages stayed longer in hospital, when compared to patients with no income or <1 minimum wage and those with >3 minimum wages; the Brazilian minimum wage in the data collection year corresponded to around US$ 200) (Table 1).

**Table 1:** Distribution of socio-demographic and lifestyle characteristics of TB patients, according to length of hospital stay and HIV status in Manaus, State of Amazonas, 2010.

	HIV positive**					HIV negative					Total				
SOCIO-DEMOGRAPHIC CHARACTERISTICS	≤25 days		>25 days		*X* ^2^	≤25 days		>25 days		*X* ^2^	≤25 days		>25 days		*X* ^2^
Sex	n	(%)	n	(%)	(P-value)	n	(%)	n	(%)	(P-value)	n	(%)	n	(%)	(P-value)
Male	57	(64.0)	39	(61.9)	0.072	58	(54.1)	25	(53.2)	0.013	115	(58.7)	64	(58.2)	0.007
Female	32	(36.0)	24	(38.1)	(0.788)	49	(45.8)	22	(46.8)	(0.907)	81	(41.3)	46	(41.8)	(0.933)
**Age group (years)**															
15–24	10	(11.2)	9	(14.3)	0.462	18	(16.8)	4	(8.5)	3.292	28	(14.3)	13	(11.8)	1.349
25–34	45	(50.5)	31	(49.2)	(0.927)	18	(16.8)	7	(14.9)	(0.349)	63	(32.1)	38	(34.5)	(0.717)
35–49	28	(31.5)	18	(28.6)		28	(26.2)	18	(38.3)		56	(28.6)	36	(32.7)	
≥50	6	(6.7)	5	(7.9)		43	(40.2)	18	(38.3)		49	(25.0)	23	(20.9)	
Mean (SD)	34.4	(0.9)	33.4	(1.1)	0.521§	45.7	(1.9)	44.4	(2.3)	0.672§	40.6	(1.2)	38.1	(1.3)	0.155§
**Race** δ															
White	14	(23.7)	10	(18.2)	3.655	12	(12.0)	5	(11.1)	4.283	26	(16.3)	15	(15.0)	4.587
Black	4	(6.8)	5	(9.1)	(0.455)	2	(2.0)	4	(8.9)	(0.369)	6	(3.8)	9	(9.0)	(0.332)
Yellow	1	(1.7)	3	(5.4)		3	(3.0)	2	(4.4)		4	(2.5)	5	(5.0)	
Brown	38	(64.4)	37	(67.3)		76	(76.0)	30	(66.7)		114	(71.7)	67	(67.0)	
Indigenous	2	(3.4)	0	(0.0)		7	(7.0)	4	(8.9)		9	(5.7)	4	(4.0)	
**Income (minimum wage)** ***															
No income or <1	10	(17.0)	13	(23.6)	**7.167**	27	(27.0)	15	(33.3)	1.225	37	(23.3)	28	(28.0)	2.060
1 to 3	32	(54.2)	37	(67.3)	**(0.028)**	63	(63.0)	24	(53.3)	(0.542)	95	(59.7)	61	(61.0)	(0.357)
>3	17	(28.8)	5	(9.1)		10	(10.0)	6	(13.3)		27	(17.0)	11	(11.0)	
**Schooling (years of study)**															
Up to 4	15	(25.4)	20	(36.4)	2.460	39	(39.0)	22	(48.9)	1.258	54	(34.0)	42	(42.0)	2.537
5 to 8	26	(44.1)	17	(30.9)	(0.292)	38	(38.0	14	(31.1)	(0.233)	64	(40.2)	31	(31.0)	(0.281)
≥9	18	(3.5)	18	(32.7)		23	(23.0)	9	(20.0)		41	(25.8)	27	(27.0)	
Mean (SD)	7.2	(0.4)	6.2	(0.5)	0.098§	5.9	(0.4)	5.1	(0.6)	0.309§	6.4	(0.3)	5.7	(0.4)	0.162§
**Postive CAGE** #															
Yes	28	(47.5)	18	(32.7)	2.566	21	(21.0)	16	(35.6)	3.459	49	(30.8)	34	(34.0)	0.285
No	31	(52.4)	37	(67.3)	(0.109)	79	(79.0)	29	(64.4)	(0.063)	110	(69.2)	66	(66.0)	(0.593)
**Fagerström (Smoking dependence)**															
Non-smoker	35	(59.3)	37	(67.3)		66	(66.0)	31	(68.9)		101	(53.4)	68	(68.0)	
Light dependence	18	(30.5)	14	(25.4)	0.121	25	(25.0)	9	(20.0)	2.180	43	(22.7)	23	(23.0)	0.599
Medium dependence	4	(6.8)	3	(5.4)	(0.941)*	8	(8.0)	3	(6.7)	(0.336)*	12	(6.3)	6	(6.0)	(0.741)*
Strong dependence	2	(3.4)	1	(1.8)		1	(1.0)	2	(4.4)		33	(17.5)	3	(3.0)	
**Drug use**															
Yes	15	(25.4)	7	(12.7)	2.946	13	(13.0)	9	(20.0)	1.181	28	(17.6)	16	(16.0)	0.112
No	44	(74.6)	48	(87.3)	(0.086)	87	(87.0)	36	(80.0)	(0.277)	131	(82.4)	84	(84.0)	(0.737)

* Refers only to information on smokers, according to dependence degree.

** HIV positive = everyone who tested positive for HIV based on the Brazilian Ministry of Health [11]. HIV positive (n = 152). HIV negative (n = 154).

*** Minimum wage in the data collection year corresponded to an average of U.S. $ 200.

δ Classification based on the Brazilian Institute of Geography and Statistics (IBGE);

§ Two tailed t-Student test, according to variance equality test.

# CAGE = Cut down/Annoyed/Guilty/Eye-opener questionnaire.

Table 2 shows the clinical and epidemiological characteristics, according to the length of hospital stay and HIV status. The following variables were associated to shorter hospitalization: positive smear test (P = 0.014), pulmonary clinical form (P = 0.011), initial 6-month treatment scheme (P = 0.029), and adverse reactions (P = 0.021).

**Table 2:** Distribution of clinical and epidemiological characteristics of TB patients, according to length of hospital stay and HIV status in Manaus, State of Amazonas, 2010.

	HIV positive**					HIV negative					Total				
CLINICAL AND EPIDEMIOLOGICAL CHARACTERISTICS	≤25 days		>25 days		*X* ^2^	≤25 days		>25 days		*X* ^2^	≤25 days		>25 days		*X* ^2^
Clinical form	n	(%)	n	(%)	(P-value)	n	(%)	n	(%)	(P-value)	n	(%)			(P-value)
Pulmonary	67	(75.3)	34	(54.0)	**9.075**	88	(82.2)	36	(76.6)	4.717	155	(79.1)	70	(63.6)	**8.638**
Extrapulmonary	14	(15.7)	23	23 (36.5)	**(0.011)**	19	(17.8)	9	(19.1)	(0.095)	33	(16.8)	32	(29.1)	**(0.013)**
Pulmonary+extrapulmonary	8	(9.0)	6	(9.5)		0	(0.0)	2	(4.3)		8	(4.1)	8	(7.3)	
**Type of discharge**															
Clinical recovery	48	(53.9)	37	(58.7)	1.344	84	(78.5)	36	(76.6)	0.070	132	(67.3)	73	(66.4)	0.704
Death	39	(43.8)	23	(36.5)	(0.511)	21	(19.6)	10	(21.3)	(0.965)	60	(30.6)	33	(30.0)	(0.703)
On request/evasion/transfer	2	(2.2)	3	(4.8)		2	(1.9)	1	(2.1)		4	(2.0)	4	(3.6)	
**Smear test**															
BAAR negative or no information	49	(55.1)	47	(74.6)	**6.057**	70	(65.4)	34	(72.3)	0.0713	119	(60.7)	81	(73.6)	**5.196**
BAARδ positive	40	(44.9)	16	(25.4)	**(0.014)**	37	(34.6)	13	(27.7)	(0.398)	77	(39.3)	39	(36.4)	**(0.023)**
**Treatment**															
Initial 6-month scheme	86	(96.6)	55	(87.3)	**4.780**	95	(88.8)	40	(85.1)	0.408	181	(92.3)	95	(86.4)	2.852
Other scheme	3	(3.4)	8	(12.7)	**(0.029)**	12	(11.2)	7	(14.9)	(0.523)	15	(7.6)	15	(13.6)	(0.091)
**Adverse reaction to drugs**															
Yes	13	(14.6)	19	(30.2)	**5.368**	29	(27.1)	29	(40.4)	2.701	42	(21.4)	38	(34.5)	**6.278**
No	76	(85.4)	44	(69.8)	**(0.021)**	78	(72.9)	28	(59.6)	(0.100)	154	(78.6)	72	(65.4)	**(0.012)**
**Hypertension and/or diabetes** *															
Yes	1	(1.1)	2	(3.2)	0.802	28	(16.2)	8	(17.0)	1.525	29	(14.8)	10	(9.1)	2.062
No	88	(98.9)	61	(96.8)	(0.370)	79	(73.8)	39	(83.0)	(0.217)	167	(85.2)	100	(90.9)	(0.151)
**Other health problems**															
Yes	42	(47.2)	39	(61.9)	3.208	62	(57.9)	28	(59.6)	0.035	104	(53.1)	67	(60.9)	1.760
No	47	(52.8)	24	(38.1)	(0.073)	45	(42.1)	19	(40.4)	(0.850)	92	(46.9)	43	(39.1)	(0.185)
**History of previous TB treatment or hospitalization**															
Yes	24	(27.0)	16	(25.4)	0.046	33	(30.8)	21	(44.7)	2.747	57	(29.1)	37	(33.6)	0.686
No	65	(73.0)	47	(74.6)	(0.829)	74	(69.2)	26	(55.3)	(0.097)	139	(70.9)	73	(66.4)	(0.407)

* Patients who reported no co-morbidities or those whose medical records contained no information were regarded as having negative co-morbidity.

** HIV positive – everyone who tested positive for HIV, based on Brazilian Ministry of Health [11].

δ BAAR = Bacilli Alcohol-Acid-Resistant.

However, there’s a need to notice the highest ratio of hospitalized patients with pulmonary TB (73.5%), but even with this burden of disease, 79.1% of patients stay in the hospital up to 25 days. Regarding the type of discharge, although 67.0% of patients showed clinical improvement, there were 30.4% of death cases. Treatment coincides with the most prevalent clinical form, thus, the initial 6-month scheme also has a higher percentage (90.2%). Adverse reactions were recorded in 26.1% of cases. The presence of HIV+ was the most frequent co-morbidity (49.7%), followed by diabetes (10.2%) and hypertension (8.5%), which, together, account for 12.7% of the co-morbidity frequency in hospitalized patients with TB.

## Discussion

Data show that the length of hospital stay of patients with TB is long, when compared to other diseases. The Brazilian Ministry of Health recommends brief hospital stay due to TB [1]. According to the findings, the average hospital stay was 28.2 days, something which confirms that this is a problem in Manaus. However, this average is much lower than that found in a study carried out in the state of Minas Gerais –64.3 days [2]. There’s a need to highlight that this is the only Brazilian study found addressing the length of hospital stay. We also have to take into account the length of hospital stay variability, as some cases require longer periods, thus influencing the average, but it wasn’t showed in that study. In the USA, a study focused on the analysis of hospital length and costs identified an average of 15 days of hospital stay [15]. Thus, *mutatis mutandis*, taking into account the Brazilian scenario, which has a universal health system, and the North American one, where health insurance companies control spending, we may regard the Brazilian length of hospital stay very high.

Surprisingly, we found out a high rate of hospitalization due to TB. In 2010, throughout Brazil, the TB hospitalization rate was 7.2 per 100,000 inhabitants, as in the state of Amazonas 12.9 cases per 100,000 inhabitants were registered. As hospitalization constitutes an analytical unit here, we assumes that it doesn’t compromise data, since only 11.1% of cases had a history of previous hospitalization. The criterion adopted for defining a hospitalization was an interval >15 days between the last discharge and the new admission; otherwise, we regarded an admission as belonging to the same hospitalization episode. Thus, the sample of patients gathered in this study may be regarded as sufficient, and this allows us to draw a profile of the length of hospital stay due to TB.

As the TB incidence is more usual among young adult men and people from lower social strata, it isn’t a matter of surprise that the same patient profile is more prone to hospitalization. Men stayed longer in hospital, perhaps due to difficulties in complying with treatment or a need for someone to remind them to take the medicine [16]. In case of worsening or complications, most men avoid seeking care [17], [18], and this leads them to postpone admission in more severe cases, requiring more time for recovery. Furthermore, we observed a higher percentage of men HIV+, something which leads us to believe that this co-morbidity strongly influences the hospitalization of patients with TB, since almost half of the study population is HIV+ (49.7%).

Age distribution doesn’t seem to have any difference regarding hospital stay. However, when we look on the data stratified by HIV positive and HIV negative hospitalized patients, it’s possible to notice that among HIV positive patients there’re much more young people than among HIV negative patients. This constitutes a major social problem, since the individual has a lot of trouble for keeping a job even in outpatient treatment and this problem worsens when he’s in need of hospitalization, especially if it’s for a long period and involves HIV treatment control. When we also take into account that TB is a severe health condition for elderly people and that these patients are more likely to worsen than to improve it, mainly due to pre-existing conditions, we would expect a higher frequency of hospitalization among elderly people, but TB/HIV co-infection is also leading to the hospitalization of young people.

There were a high ratio of patients who earn from 1 to 3 minimum wages (51.0%) and, out of these, 31% remain hospitalized for less than 25 days. Race depicts the distribution of this variable among the population, as the significant mixing doesn’t allow us to infer that brown-skinned people would be at greater risk of hospitalization, but it only shows that race mixing is much larger in local population, according to the Brazilian race distribution, where there’s a high rate of miscegenation [19].

Schooling, a socioeconomic status indicator, shows that those with higher education stayed less in hospital, when compared to those with lower education among HIV+ patients (P = 0.098) and HIV− patients (P = 0.309). Although the differences aren’t significant, they may prove the individual’s ability to understand the treatment, since the patient who has more information can better cooperate to the treatment [20], [21]. Similarly, the findings corroborate other studies which identified a higher risk for hospitalization and its lengthen among people from lower social strata [22].

About 1/3 of patients were classified as alcoholic people. The strong relationship between alcoholism and TB is widely known. TB has a worse prognosis in alcoholic patients when compared to non-alcoholic patients [23]. Although this study hasn’t found any significant association, we may regard this figure as underestimated, because although there’s information indicating that drinking started six months before hospitalization, it’s known that patients tend to confuse it with alcoholism during hospitalization and deny alcohol consumption. Some bias may also be the reason for the omission of such information, since this behavior is socially criticized. Therefore, there’s a need for further studies addressing this aspect.

The consumption of cigarettes wasn’t associated to the length of hospital stay. However, the highest percentage of hospitalized patients lays on the mild dependence degree, but it apparently doesn’t influence the length of hospital stay. Smoking must be investigated as a predictor of hospitalization rather than a factor specifically influencing its length [24], [25], or perhaps as a risk factor for the development of active disease, since it adversely affects the lungs and decreases the individual’s immunity.

Pulmonary TB was prevalent and, apparently, this clinical form has the shortest length of hospitalization. Although there’s no significant association, we must consider that pulmonary TB forms are known to have transmission potential [26] and, this way, keeping patients with pulmonary TB in hospitals may increase the risk of spreading TB in the hospital environment, especially if the hospital doesn’t have a proper structure for providing TB patients with treatment [1]. Besides, we expect that extrapulmonary TB forms, whose treatment is more difficult, would account for most of hospitalization cases.

When we observe TB treatment, it’s possible to verify that more than 80% of cases adopted the initial 6-month scheme, used to treat the pulmonary TB form. However, when we observe the distribution of other schemes, it’s possible to note that this occurred among HIV positive patients who need long hospitalization periods. This difference doesn’t occur in HIV negative patients. Of course, treatment isn’t responsible for the length of hospital stay, but we may understand that this stresses the severity of cases, especially those among HIV positive patients. This indicates that HIV positive patients need an alternative scheme and perhaps this has an influence on the length of hospital stay.

The most frequent type of discharge was clinical recovery, with no association to the length of hospital stay. However, we observed that, in cases of death, people spent less time in hospital (19.6%). This fact indicates that the number of deaths due to TB is considerable, perhaps because it’s a more severe condition when patients come to the hospital. A limiting factor in this analysis was that we didn’t measure the disease stage, either TB or HIV, since this study has a cross-sectional design. However, we assume that this fact isn’t considerable, since there was no difference in death cases according to HIV positivity and the length of hospital stay.

The criteria for admission and discharge were based on the patient clinical status, according to the clinical evaluation, as indicated in the medical record. It’s worth pointing out that the Brazilian National TB Control Program sets up the recommendations for hospitalization to be adopted in the whole country [1]. Regarding discharge, the Program regards as suitable a hospitalization period as short as possible [1]. Thus, these findings are important because it was possible to show that patients with TB have stayed hospitalized for a long time, something which reflects a failure in primary care, where TB cases should be treated.

Negative smear test or no information is more usual in those who remain for more than 25 days in hospital. This is already expected, since those with negative smear tests tend to stay longer in hospital for diagnostic investigation.

The most usual treatment is the initial 6-month scheme, since this is the scheme used for pulmonary TB. However, it’s noteworthy that 13.6% of those using other schema stayed longer in hospital. Although this association wasn’t significant, it must be investigated in further studies on multidrug resistance. We must depict that all hospitals adopted the standardized TB treatment as initial 6-month treatment scheme, using rifampicin, isoniazid, pyrazinamide, and ethambutol, as recommended by the World Health Organization [27]. Table 2 shows that almost 90.0% of patients adopted the initial 6-month treatment scheme.

The occurrence of adverse reactions is an indication for hospitalization [1]. However, this was the case in only 26.1% of hospitalizations studied. Furthermore, there’s a need for finding out that this event is more frequent in HIV+ cases. It instigates studies regarding other causes of hospital admission and related factors, as well as this study on the length of hospital stay. We should notice whether this fact is being adequately investigated and treated, in order to identify the adverse drug reactions and the best way to deal with them. It may also be an indicator whether the reactions are mild, so that the patient can be treated on an outpatient basis.

Regarding co-morbidities, the most frequent one was HIV-related (49.7%) and, at very close percentages, hypertension (8.5%) and diabetes (6.8%). No co-morbidity was significantly associated to the length of hospital stay. It’s known, however, that in many cases such co-morbidities lead to hospitalization, as the patient would be primarily admitted not due to TB, but to treat a related factor, which may have worsen during TB treatment.

The collection of primary data, focused on the hospitalization of patients with TB, a careful surveillance system, in order to detect 100% of admissions, the sample size, and the scope of referral hospitals in Manaus are the strong aspects of this study. This allowed us to develop a detailed examination of individual factors associated to the length of hospital stay in a city with an extremely high TB incidence rate.

The length of hospital stay of patients with TB is subject to factors which are intrinsic to the individual and service-related. However, this analysis focuses on factors related to the individual and, therefore, this constitutes a limitation of this study, which wasn’t designed with a focus on service. Thus, the socio-demographic, clinical, and epidemiological elements identified allow us to detect factors related to the length of hospital stay, without interfering with the findings, and they suggest a service review in further studies.

Knowing the limiting factors which lead patients to undergo longer hospitalizations allows us to tackle problems or, at least, work along with patients according to the identified profile. This way, we can contribute to their earliest recovery, without ignoring the simultaneous role played by the TB/HIV co-infection.
